# Drought and child mortality: a meta-analysis of small-scale surveys from Ethiopia

**DOI:** 10.1038/s41598-017-02271-5

**Published:** 2017-05-19

**Authors:** Tefera Darge Delbiso, Chiara Altare, Jose Manuel Rodriguez-Llanes, Shannon Doocy, Debarati Guha-Sapir

**Affiliations:** 1 Centre for Research on the Epidemiology of Disasters (CRED), Institute of Health and Society, Université catholique de Louvain, Brussels, Belgium; 2 Research and Analysis Unit, Action Against Hunger, Paris, France; 3 Food Security Unit, Sustainable Resources Directorate, European Commission Joint Research Centre, Ispra, Italy; 4 Center for Refugee and Disaster Response, Johns Hopkins Bloomberg School of Public Health, Baltimore, USA

## Abstract

Despite the intuitive connection between drought and mortality, we still lack a sound quantitative synthesis of evidence drawn from the available data. In this study, we estimate the pooled under-five death rates (U5DR) and assess the effect of drought on child death in Ethiopia. Small-scale mortality surveys were searched from the Complex Emergency Database and then aggregated spatially and temporally with drought exposure data from the Global Drought Monitor and food insecurity data from the Famine Early Warning Systems Network. A Bayesian Poisson meta-analysis was performed on 88 surveys conducted in Ethiopia between 2009 and 2014, consisting of 55,219 under-five children. The pooled U5DR was estimated at 0.323/10,000/day (95% credible interval, CrI: 0.254–0.397), which is below both the emergency and the baseline death rate thresholds of sub-Saharan Africa. We failed to find a plausible association between drought and U5DR. However, minimal food insecure areas showed elevated U5DR compared to stressed food insecure areas. Furthermore, the U5DR increases as the prevalence of acute malnutrition increases. Targeted interventions to improve the underlying causes of child malnutrition are crucial. Further, revising and updating the existing mortality thresholds, both the baseline and the emergency, is recommended.

## Introduction

Humanitarian emergencies, which are mainly triggered by either civil conflicts or natural disasters, undermine the livelihood and health of the affected population and increase mortality^1^. Drought is a natural and cyclical event that develops slowly, worsens gradually, and results in destitution, starvation and death if not addressed properly. Between 1994 and 2013, more than one billion people were affected by drought worldwide. Africa, accounting for 41% of all drought events, suffered more frequent droughts than any other continent^2^. The health impact of drought is complex and long lasting. Drought has been associated with excess mortality through inducing nutrition and health problems, such as undernutrition, micronutrient deficiencies, food- and water-borne diseases; aggravating chronic diseases; declining crop and livestock production; contributing to inflation of food prices; and triggering drought-induced migration. When combined with poverty, weak health infrastructure and poor sanitation, its impact is even worse^3, 4^.

Ethiopia is prone to weather-related shocks, such as droughts and floods. As a result, the country has been experiencing recurrent droughts and food shortages since the 1950s. More than 80% of its population are dependent on subsistence and rain-fed agriculture, meaning that the majority of the population is highly vulnerable to drought^5^. The most severe of which was the mid-1980s famine, in which, even with a massive humanitarian response, elevated malnutrition prevalence was observed and an estimated 700,000 excess deaths occurred^6, 7^. Despite the noticeable improvements in the overall development and the achievement of the Millennium Development Goals (MDGs) target of reducing hunger by half between 1990 and 2015^8^, every year, a significant portion of Ethiopians rely on food assistance mainly due to recurrent droughts. For example, in 2015–16, following the devastating impact of El Niño weather events, Ethiopia experienced one of the worst droughts in the last five decades. Consequently, more than 10 million people (over 10% of the total population) were in need of urgent food assistance^9^.

Pooled data investigating the effects of drought on child mortality are scarce in crisis-affected areas. A recent systematic review by Stanke pointed out the lack of sound quantitative syntheses on the drought-mortality link, possibly due to the differences between studies in regard to settings, methods and outcomes measured^3^. The few available studies have focused on the immediate causes of malnutrition and mortality in complex emergencies, which include inadequate dietary intake and diseases, such as diarrheal diseases, acute respiratory infections, measles, malaria, and severe malnutrition^10, 11^. Only one study investigated the relationship between drought and child survival in Ethiopia and this was conducted in the 2002/03 drought^12^. No difference in child mortality was observed between drought affected and non-affected areas; however, study limitations, such as not considering drought intensity, and the one-year time elapse between the drought occurrence and when the study was conducted may have contributed to null findings^12^. To address this research gap and provide a sound quantitative synthesis, we explored the effect of drought on child mortality in Ethiopia between 2009 and 2014 considering real-time data on the intensity of drought exposure. The specific aims of the study were to provide a pooled estimate of the under-five death rates (U5DR) and to investigate the effect of drought on child death while controlling for the possible contextual factors, identified based on the United Nations Children’s Fund (UNICEF) conceptual framework of malnutrition^13, 14^ and available data. These factors include acute malnutrition, measles-containing vaccination (MCV), food insecurity, and livelihood zones.

## Results

### Identification and selection of surveys

**Figure 1:**
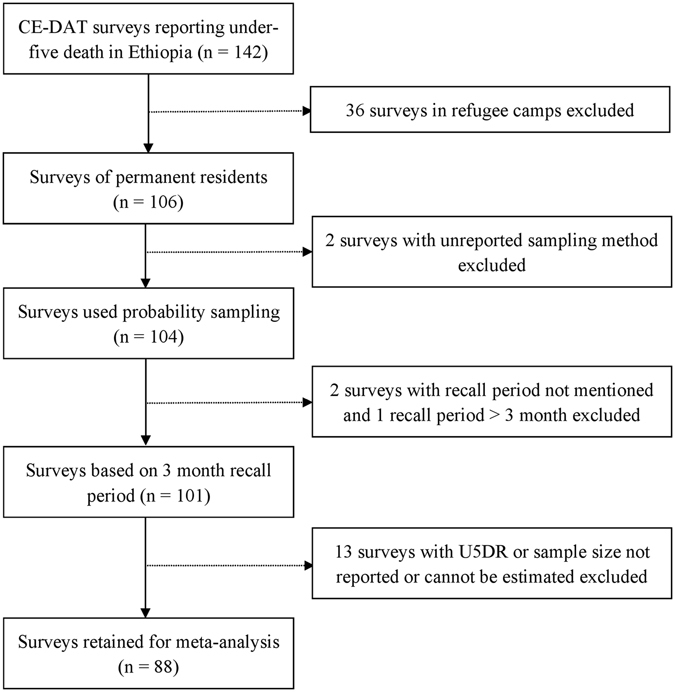
Flowchart of the small-scale mortality surveys included in the meta-analysis from Ethiopia, 2009–2014. CE-DAT: Complex Emergency Database. USDR: Under-five Death Rate.

We initially extracted 142 small-scale surveys reporting under-five deaths from the Complex Emergency Database (CE-DAT). The surveys were conducted between January 2009 and December 2014 by humanitarian agencies operating in Ethiopia. The following inclusion criteria were applied to select surveys for the final meta-analysis: 1) surveys conducted in permanent resident populations; 2) surveys based on probability sampling methods (simple random sampling, systematic sampling, or cluster sampling); 3) surveys based on 3 month recall period; and 4) the U5DR and/or sample size is provided or can be estimated. We excluded surveys conducted in refugee camps, sampling methods not indicated, and recall period not reported. After exclusions, we identified 88 surveys to be included in the meta-analysis (Fig. 1). The number of children per survey ranges from 239 to 1007. More than a third of the surveys were conducted in 2010 (n = 31, 35.2%), while n = 4 (4.5%) surveys were conducted in 2014. The details of each of the surveys are available in Supplementary Table S1.

### Description of the surveys

**Figure 2:**
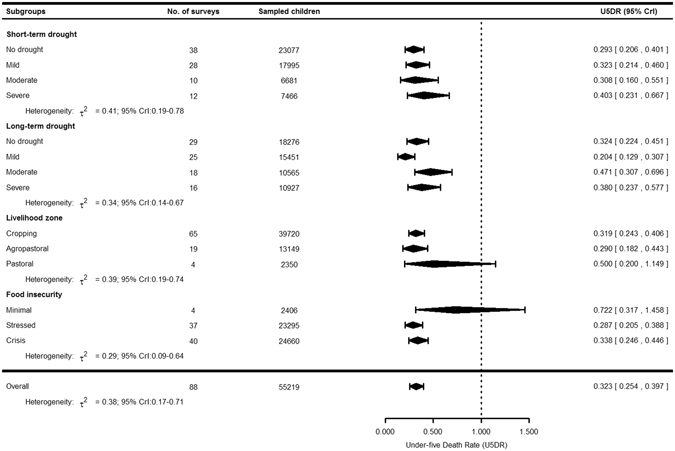
Forest plot showing the pooled U5DR and their 95% CrI’s, stratified by short- and long-term drought exposure levels, livelihood zones and food insecurity level from 88 small-scale mortality surveys from Ethiopia, 2009–2014. The U5DR was based on the small-scale retrospective household surveys^32^. The short- and long-term droughts in a given location were based on the 3- and 12-month standardized precipitation evapotranspiration index (SPEI), respectively^35^. Food insecurity was based on the integrated food security phase classification (IPC)^38^; and it is available for 81 survey locations. Livelihoods are based on similarities in terms of economic activity, food source, and income and market opportunities. CrI: credible interval.

The 88 surveys included in the meta-analysis consisted of a sample of 55,219 children under-five years of age. The surveys covered 7 of the 11 administrative regions in Ethiopia. Most of the surveys were from the three highly populated regions of the country: Oromia (n = 38, 43.2%), Amhara (n = 22, 25.0%), and the Southern Nations, and Nationalities and People (n = 18, 20.5%) (Table 1). Regarding drought exposure, n = 50 (56.8%) and n = 59 (67.0%) of the surveys were conducted in areas affected by mild to severe drought in the short- and long-term, respectively. Most of the surveys (n = 65, 73.8%) were conducted in cropping areas (versus pastoral and agropastoral). Moreover, n = 84 of the surveys (95.1%) were conducted in areas suffering from stressed or crisis level food insecurity (Fig. 2).

**Table 1:** Description of the 88 small-scale mortality surveys from Ethiopia, 2009–2014.

Administrative regions	Number of surveys (%)	Sampled children (%)
Oromia	38 (43.2%)	26190 (47.4%)
Amhara	22 (25.0%)	11119 (20.1%)
SNNP	18 (20.4%)	12144 (22.0%)
Afar	5 (5.7%)	2975 (5.4%)
Benishangul-Gumuz	2 (2.3%)	1110 (2.0%)
Dire Dawa	2 (2.3%)	1093 (2.0%)
Gambela	1 (1.1%)	588 (1.1%)
Total	88 (100%)	55219 (100%)

SNNP: Southern Nations, Nationalities and People.

### Pooled U5DR across the surveys, subgroup meta-analysis and meta-regression

The forest plot in Fig. 2 shows the pooled U5DR according to the subgroups. The pooled U5DR in the study period was 0.323/10,000/day (95% credible interval, CrI: 0.254–0.397). The heterogeneity among the survey estimates was *τ* ^2^ = 0.38 (95% CrI: 0.17–0.71) (Fig. 2). The U5DR was not associated with both short- and long-term droughts (Table 2). Pastoral areas showed higher U5DR (0.500; 95% CrI: 0.200–1.149) (Fig. 2), but there was no plausible difference compared to agropastoral areas (Table 2) which had U5DR of 0.290 (95% CrI: 0.182–0.443) (Fig. 2). Minimal food insecure areas had a higher U5DR (0.722; 95% CrI: 0.317–1.458) than areas reporting stressed food insecurity (U5DR = 0.287; 95% CrI: 0.205–0.388) (Fig. 2) and the difference was statistically plausible (DRR = 2.36; 95% CrI: 1.05–5.02 Model 1; DRR = 2.36; 95% CrI: 1.09–4.68 Model 2) (Table 2). Every one-percent increase in the prevalence of global acute malnutrition (GAM) was associated to an increase of 9% (95% CrI: 4–14%) and 7% (95% CrI: 3–12%) in the median U5DR in Model 1 and Model 2, respectively (Table 2). On the other hand, there was no plausible association between U5DR and both MCV coverage and livelihood zones. Further, the interaction between droughts and the moderators were implausible. The inclusion of moderators in the meta-regression explained the heterogeneity substantially – the *τ* ^2^ statistic decreased by 50.0% and 65.9% for Model 1 and Model 2, respectively (Table 2).

**Table 2:** Results from a ﻿Bayesian Poisson meta-regression models of small-scale mortality surveys from Ethiopia, 2009–2014.

Moderators	Model 1		Model 2	
	Posterior DRR	95% CrI	Posterior DRR	95% CrI
**Drought exposure**				
No drought	Ref			
Mild drought	0.97	0.61; 1.54	0.76	0.44; 1.28
Moderate drought	1.37	0.69; 2.63	1.62	0.98; 2.71
Severe drought	1.27	0.73; 2.26	1.20	0.71; 2.01
**Food insecurity**				
Minimal	2.36	1.05; 5.02	2.36	1.09; 4.68
Stressed	Ref			
Crisis	1.08	0.72; 1.63	1.14	0.77; 1.69
**Livelihood zones**				
Cropping	1.04	0.62; 1.74	1.10	0.67; 1.82
Agropastoral	Ref			
Pastoral	1.25	0.45; 3.45	1.66	0.64; 4.39
**Prevalence of GAM**	1.09	1.04; 1.14	1.07	1.03; 1.12
**MCV coverage**	1.00	0.99; 1.01	1.00	0.99; 1.01
**Heterogeneity:** *τ* ^2^ (95% CrI)	0.19 (0.01; 0.54)		0.13 (0.01; 0.44)	

Model 1 investigates the effect of short-term droughts on child mortality, whereas Model 2 investigates the effect of long-term droughts on child mortality, both adjusted for survey-specific moderators (food insecurity, livelihood zones, prevalence of GAM, and MCV coverage). The prevalence of GAM was calculated based on the weight for height z-score index^34^. The MCV coverage was based on vaccination cards and/or caregiver’s recall. The statistical significance is based on the non-inclusion of 1.0 in the 95% CrI. DRR: death rate ratios; CrI: credible interval; Ref: reference category.

## Discussion

Child mortality reflects the general health of the population and is one of the indicators used to benchmark the severity of a crisis^11, 15^. Despite anecdotal and crude evidence on the association between drought and mortality in Ethiopia and elsewhere in the world^3, 6^, rigorous synthesis of empirical data is lacking. This could be partly due to the unclear definition of the onset and ending of a drought, its long lasting cumulative effect, and poor record-keeping systems^2–4^. With the aim of addressing this gap, we conducted a meta-analysis of 88 small-scale mortality surveys (consisting of 55,219 under-five children) from areas affected by humanitarian crises in Ethiopia between 2009 and 2014.

Overall, the pooled U5DR in the study period was 0.323/10,000/day, which is considerably lower than both the emergency threshold death rate of 2.1/10,000/day and the baseline threshold of 1.07/10,000/day for sub-Saharan Africa^16^. Ethiopia, in general, has shown great improvement in reducing child mortality and successfully achieved the MDG target of two-thirds reduction in under-five mortality rate between 1990 and 2015^8^. Consequently, the under-five mortality rate dropped from 166 deaths per 1000 live births in 2000 to 67 in 2016^17^. This achievement could be attributed to the progress made in improving the coverage of health services and immunization^8, 18^.

Crisis-affected areas often show elevated mortality. For example, fragile states in Africa reported 20% higher child mortality compared to low-income countries^19^. However, we obtained a pooled U5DR estimate, which is even lower than the country’s average of 0.4/10,000/day, calculated based on the mortality data of UNICEF and the population data of the United Nations population division. Moreover, only 2.3% of the surveys had U5DR above the baseline threshold and no survey had U5DR around the emergency threshold of sub-Saharan Africa. This may reflect the fact that mortality is lower in areas where humanitarian agencies are operational, as their support to national programmes contributes to improving health. Furthermore, security conditions often prevent agencies from accessing some of the severely affected zones. Finally, possible underestimation of mortality, due to inaccessibility of some affected areas mentioned above and survival bias owing to the possible disappearance of the entire households in crisis-affected areas^20^, should not be ruled out. Overall, the lower than expected U5DR in our study raises questions regarding the accuracy of mortality thresholds being used, since substantial reduction in child mortality has been achieved in the MDG era^21^. Our findings highlight the need to review and update the existing mortality thresholds^16^, both the baseline and the emergency thresholds.

Neither short- nor long-term droughts were associated with U5DR. This could be explained by the resilience capacity the country developed over time to deal with shocks related to drought and food shortage. First, the country adopted a national policy on disaster prevention and management in 1993 to reduce disaster risks and its potential consequences^22^. Weather forecasting and early warning systems have been strengthened, and relief assistance has become more predictable and timely^5^. Second, in 2005, Ethiopia launched a safety net program targeting chronically food insecure households in famine-prone areas to provide a long-term solution for minimizing vulnerability to food insecurity. The program has been providing assistance in the form of food and cash transfer, building resilience to climate-related impacts, improving access to health, and enhancing risk financing mechanisms to protect assets from being eroded by recurrent shocks. The program benefits approximately 8 million people in rural areas annually^8, 23^. Third, road network development over the last decade has helped farmers easily access markets and relief providers reach remote villages. Fourth, the humanitarian response has become more efficient as it is based on early warning and assessment results during emergency^22^.

The meta-regression shows that U5DR increases with the prevalence of GAM, echoing the well-documented fact that child wasting and mortality are directly associated^11, 24–26^. Malnutrition aggravates infectious diseases, thereby increasing the risk of child mortality^27^. For example, in 2011, about 45% of all child deaths in the world (3.1 million child deaths) were attributable to undernutrition^24^; and in Ethiopia, this figure was 28% between 2004 and 2009^28^. The effect is more apparent in countries going through humanitarian crises where the prevalence of malnutrition is twice that of low-income countries^19^. Despite the declining trend, the prevalence of acute malnutrition in crisis-affected areas in Ethiopia remains high, more than 10%^26, 29^. Therefore, unless continuous and targeted interventions are identified and implemented to improve child nutrition, a significant number of child deaths will occur due to acute malnutrition.

Food insecurity can be both a cause and a consequence of protracted crises and it increases child mortality by affecting food intake and increasing susceptibility to diseases^30^. Surprisingly, our result shows that areas with minimal food insecurity had higher U5DR than areas with stressed food insecurity. This finding contradicts the existing evidence^30, 31^, which indicates that higher food insecurity is directly associated with higher child mortality. One possible explanation is that areas with minimal food insecurity may not attract the attention of the government and aid agencies. Thereby, they will not receive the assistance which contributes to the improvement of child survival, such as health care, food assistance and other relief commodities. In this case, households may be forced to rely on their own livelihoods without external aid, thus there is a need to promote resilience within these households and communities. In a sense, these findings can be interpreted as a sign of the continued high reliance on aid by rural households. Nonetheless, this result needs to be interpreted with caution. First, we do not have full information on the possible contextual factors in the surveys (e.g., disease incidence, breastfeeding, access to health) which could elevate U5DR. Second, the reliability of the estimates might be affected as we have only four surveys conducted in minimal food insecure areas. Thus, further research with a substantial number of surveys and considering the full range of contextual factors is warranted.

While interpreting the results, the following limitations should be taken into consideration. First, retrospective mortality surveys are heavily affected by recall bias which may lead to under reporting of deaths or reporting of incorrect dates. Thus, to reduce the recall bias, we limited our study to surveys with a short recall period (3 months). Second, despite improvements in data quality assurance of mortality indicators in CE-DAT, and despite CE-DAT is a unique and detailed source of nutrition and mortality data for crisis-affected areas, the quality of survey implementation cannot be verified in-depth^32^. Third, although CE-DAT strives to gather all nutrition and mortality surveys from humanitarian agencies, the possibility of missing some of the surveys is inevitable. However, we believe that this number is insignificant for Ethiopia, as the CE-DAT project has a strong data sharing agreement with the majority of the agencies operating in the country. Fourth, the majority of the small-scale surveys in CE-DAT does not have information on the underlying causes of child mortality, such as disease incidence, child care practices, water and sanitation coverage and non-food interventions, which could account for part of the unexplained variation in the data. Despite these limitations, our meta-analysis sheds light on the limited literature surrounding the topic and stimulates debates for further exploration of the link between drought and child mortality in humanitarian emergencies.

In conclusion, the estimated pooled U5DR was below both the country’s average and the baseline threshold for sub-Saharan Africa. This indicates the need to review and update the existing mortality thresholds. Exposure to both short-and long-term droughts was not associated with U5DR in Ethiopia. However, minimal food insecurity was associated with elevated U5DR and hence, these areas should not be overlooked by the intervention programs. Further, targeted interventions to improve the underlying causes of child malnutrition in crisis-affected areas are crucial, as acute malnutrition increases U5DR. Finally, our study recognizes the enormous progress made by the country in enhancing drought resilience.

## Methods

### Under-five death, nutrition, and vaccination data

In the absence of functioning vital registration systems in crisis-affected areas where an elevated prevalence of acute malnutrition and mortality rates are of concern, mortality information is often gathered through retrospective household surveys^15^. Among them are small-scale operational surveys, which are conducted by humanitarian organizations in crisis-affected areas. Small-scale surveys have been shown to be a useful source of information for humanitarian decision making^32^. These surveys are publically available in the CE-DAT, www.cedat.be. For the current study, we used surveys conducted in Ethiopia between January 2009 and December 2014. The timeframe for the study was based upon data availability for the contextual factors considered in our analytical framework.

The CE-DAT mainly compiles surveys on nutrition, mortality and vaccination coverage from humanitarian organizations, health ministries and UN agencies. Most of the included surveys follow a standardized methodology for nutrition and mortality surveys, the Standardized Monitoring and Assessment of Relief and Transitions (SMART) methodology^33^. Within Ethiopia, the survey process is monitored by the Emergency Nutrition Coordination Unit. The survey reports are further validated by the CE-DAT team using the established completeness checklist, prior to entering the survey results into the database^32^. The following indicators were extracted from the CE-DAT: the number of under-five deaths, the number of under-five children and the U5DR; the prevalence of GAM among children 6–59 months of age, also referred to as wasting; the MCV coverage among children 9–59 months of age; the survey coverage area (the third administrative level, ‘*woreda*’) in addition to the month and year the survey took place (see the Supplementary Table S1).

The U5DR reported in the CE-DAT, expressed as deaths per 10,000 children per day (i.e., deaths/10,000/day), were calculated using the number of deaths in children under-five years of age, the mid-year population of under-five years at risk of death, and the recall period. The prevalence of GAM was calculated based on z-scores of weight and height measurement of children aged 6–59 months. The weight-for-height z-score below −2 is indicative of GAM or wasting^34^. The MCV coverage was based on vaccination cards. If this was not available, it was based on caregiver’s recall. Then it was calculated as the number of children vaccinated against measles over the total number of children aged 9–59 months eligible for vaccination.

### Drought exposure data

Different indices have been developed to quantify, monitor and analyse drought (e.g., the Palmer drought severity index, the standardized precipitation index, and the standardized precipitation evapotranspiration index)^35^. We used the Standardized Precipitation Evapotranspiration Index (SPEI), which is an improved index to identify the spatial and temporal extent of drought exposure and its intensity. The SPEI is calculated for different time-scales (e.g., 1-, 3-, 6-, 12-month) considering the combined effect of precipitation and temperature. For example, a 3-month SPEI obtained at the end of August compares the precipitation and temperature total of June, July and August period with the same 3-month period over historic records (i.e., 1950 onwards). Shorter time-scales (e.g., 3- and 6-month SPEI) identify more frequent droughts of shorter duration affecting agricultural practices, whereas longer time-scales (e.g., 12- and 24-month SPEI) detects less frequent but longer-lasting droughts associated with water resource^35, 36^. The different time-scales are therefore important for assessment of drought effects on child mortality, as the effects are likely to vary with the frequency and duration of droughts. Thus, in this study, the 3-month and 12-month SPEIs were used to identify the short- and long-term droughts, respectively.

The SPEI data was downloaded from the Global Drought Monitor, http://sac.csic.es/spei/map/maps.html, which provides robust information about drought conditions at the global-scale with 0.5 degree spatial resolution and monthly time resolution. The SPEI values for a specific location and month were extracted based on the starting time and the centroid of the survey coverage area. The SPEI takes positive or negative values, typically in the range between −2.5 and +2.5, indicating wet- or dry-conditions, respectively^36^. For ease of interpretation, we categorized the SPEI values into: no drought (SPEI > 0), mild drought (−1 < SPEI ≤ 0), moderate drought (−1.5 < SPEI ≤ −1) and severe to extreme drought (severe drought hereafter) (SPEI ≤ −1.5)^37^.

### Food security and livelihood zones data

Food security encompasses availability, accessibility, utilization and stability of food for a healthy and active life^30^. We obtained the food security data from the Famine Early Warning Systems Network (FEWS NET, http://www.fews.net/), which provides evidence-based analysis on early warning and food insecurity. FEWS NET uses the Integrated Food Security Phase Classification (IPC), a set of tools and procedures which serve to identify and classify the severity of food insecurity. The IPC is based on the four pillars of food security analysis: accessibility, availability, utilization and stability, and it classifies areas with food insecurity into five phases – minimal, stressed, crisis, emergency, and famine^38^. We gathered historical food security phase data from the FEWS NET quarterly food security reports. Data was available for 81 survey areas from 2009 onwards. In addition, we obtained the livelihood zone classification data (cropping, agro-pastoral and pastoral) from FEWS NET. Livelihoods are based on similarities in terms of economic activity, food source, and income and market opportunities. The food security phase and livelihood zones data of a given survey coverage area were then matched with CE-DAT data using the GPS coordinate.

### Statistical analysis

For surveys that did not report the number of under-five deaths, it was calculated by multiplying the mortality rate with sample size and recall period. The datasets were merged across space and time and then random-effects meta-analysis model was fitted to summarize survey results and explore heterogeneity across independently conducted but methodologically similar small-scale surveys. Summarizing data with this approach increases statistical power to detect effects, and improve precision^39^. When applied in humanitarian decision making, the analytical approach can strengthen informed response and preparedness thereby enhancing evidence-based decision making at large scale^26, 29, 32^.

We explored the variability across the survey estimates using the *τ* ^2^ statistic (the between-survey variance). To explain the variability, meta-analyses were performed on subgroups based on survey-specific contextual factors or moderators (drought exposure levels, food insecurity, and livelihood zones); and subsequently a meta-regression was fitted adjusting for additional moderators, prevalence of GAM and MCV coverage. We also tested the interaction effects to assess whether the association between drought and U5DR varied by the moderators. Among the 88 surveys included in our meta-analysis, 16 (18.2%) had zero counts for the number of under-five deaths. Using the inverse-variance methods for such data is problematic because the estimates and their variances are not defined for zero death counts. Hence, Poisson regression model for meta-analysis was used as a remedy to circumvent these issues^40^. The model was then fitted in a Bayesian framework in an attempt to incorporate all uncertainties in estimating the parameters and to benefit from its flexibility for inference^41^.

Assume that the observed number of under-five deaths in survey *i*, *d*_*i*_(*i* = 1 ,…, 88), follows a Poisson distribution with *λ*_*i*_ (expected number of under-five deaths in survey *i*). A random-effects meta-analysis using Poisson regression is then expressed as:

1 $$\begin{array}{c}{d}_{i} \sim Poisson({\lambda }_{i})\\ \mathrm{log}({\lambda }_{i})={\mu }_{i}+{\beta }_{i}{x}_{i}+\,\mathrm{log}({n}_{i})\\ {\mu }_{i} \sim Normal(\theta ,\,{\tau }^{2})\end{array}$$

where *μ*_*i*_ represents the under-five death rate in survey *i*, *θ* represents the pooled under-five death rate, *τ* ^2^ is the between-survey variance, ${x}_{i}^{^{\prime} }s$ are the survey-specific moderators, ${\beta }_{i}^{^{\prime} }s$ are the regression coefficients, and the number of under-five children in survey *i*,*n*_*i*_, is included as log offset in the model. The unknown parameters were assigned vague priors to allow the results to be based on the data: *inverse*−*gamma*(0.001,0.001) for the variance parameter, *τ* ^2^; and *Normal*(0,10^6^) for *θ* and ${\beta }_{i}^{^{\prime} }s$. The Bayesian meta-analysis was performed in WinBugs version 1.4^42^, where the Markov Chain Monte Carlo (MCMC) sampling is used to make inference. We run the Markov chain for 50,000 iterations after a start-up run of 10,000 iterations. We then assessed the convergence of the chain visually inspecting the trace- and autocorrelation-plots^41^. The *metafor* package^43^ in R statistical software^44^ was used to generate the forest plot.

Two meta-regression models were fitted based on the duration of droughts: Model 1 investigating the effect of short-term droughts on child mortality, while Model 2 focused on the long-term droughts, both adjusted for the survey-specific moderators. Finally, the median of the posterior distribution was calculated to provide estimates of the U5DR, death rate ratios (DRR), and 95% credible intervals (CrI) derived from the 2.5^th^ and 97.5^th^ percentile. The statistical significance (plausibility) was judged based on the non-inclusion of 1.0 in the 95% CrI.

## Electronic supplementary material

Supplementary Table S1
