# Supplementary material for: Drought and child mortality: a meta-analysis of small-scale surveys from Ethiopia
Source: Sci Rep. 2017 May 19;7:2212. doi: 10.1038/s41598-017-02271-5 (PMC5438405; doi:10.1038/s41598-017-02271-5)
Supplement: Supplementary file 1 — Supplementary Table S1 [file 41598_2017_2271_MOESM1_ESM.pdf]

Drought and child mortality: a meta-analysis of small-scale surveys from Ethiopia

Tefera Darge Delbiso, Chiara Altare, Jose Manuel Rodriguez-Llanes, Shannon Doocy and Debarati Guha-Sapir

Supplementary Table S1: Details of data used in the meta-analysis

Survey ID	Survey area (admin1 - admin2 - admin3)	Month-Year	No. of deaths	No. of children	Short-term drought	Long-term drought	GAM prevalence	MCV coverage	Food insecurity	Livelihood zones
AM01	Amhara - South Wollo - Ambasel	03 - 2009	2	288	moderate	moderate	11.1	87.5	crisis	cropping
AM02	Amhara - South Wollo - Ambasel	08 - 2009	0	347	normal	moderate	12.5	82.2	crisis	cropping
AM03	Amhara - South Wollo - Dessie Zuria	09 - 2009	2	239	mild	moderate	14.2	83.4	crisis	cropping
AM04	Amhara - North Wollo - Gubalafto	06 - 2009	1	292	severe	moderate	8.4	78.9	crisis	cropping
AM05	Amhara - South Wollo - Wara Babo	09 - 2009	5	422	mild	moderate	11.1	83.6	crisis	cropping
OR01	Oromia - West Hareghe - Boke	03 - 2009	7	780	mild	moderate	14.8	32.6	crisis	agropastoral
OR02	Oromia - West Harerghe - Burka Dintu	07 - 2009	8	820	moderate	moderate	14.4	18.5	crisis	agropastoral
OR03	Oromia - East Harerghe - Deder	09 - 2009	1	766	moderate	moderate	10.6	54.7	crisis	cropping
OR04	Oromia - East Harerghe - Goro Gutu	11 - 2009	1	764	mild	severe	7.5	61.0	crisis	cropping
OR05	Oromia - West Harerghe - Hawigudina	07 - 2009	2	802	mild	mild	12.3	24.4	NA	agropastoral
OR06	Oromia - East Harerghe - Kersa	03 - 2009	2	678	mild	normal	14.8	64.2	crisis	cropping
OR07	Oromia - East Harerghe - Meta	09 - 2009	2	764	severe	severe	11.4	61.9	crisis	cropping
SN01	SNNP - Gamo Gofa - Arba Minch Zuria	01 - 2009	0	702	normal	normal	1.4	92.8	NA	cropping
SN02	SNNP - Sidama - Boricha	04 - 2009	5	706	moderate	mild	11.2	68.1	crisis	cropping
SN03	SNNP - Gedeo - Bule	02 - 2009	2	996	mild	normal	7.4	62.1	NA	cropping
SN04	SNNP - Gedeo - Bule	09 - 2009	3	655	severe	moderate	4.4	27.2	crisis	cropping
SN05	SNNP - Wolayita - Damot Gale	03 - 2009	8	733	mild	normal	9.9	64.2	NA	cropping
SN06	SNNP - Gedeo - Gedeb	09 - 2009	2	700	severe	moderate	6.2	40.3	crisis	cropping
SN07	SNNP - Hadiya - Shashago	01 - 2009	1	591	normal	normal	8.3	10.2	NA	cropping
SN08	SNNP - Sidama - Shebdino	03 - 2009	3	766	mild	normal	17.5	68.9	stressed	cropping
SN09	SNNP - Hadiya - Soro	01 - 2009	4	524	normal	normal	14.3	64.3	NA	cropping
AM06	Amhara - Wag Hamra - Abergele	12 - 2010	2	542	severe	moderate	11.9	75.8	crisis	cropping
AM07	Amhara - South Wollo - Ambasel	02 - 2010	0	256	normal	mild	8.6	87.5	crisis	cropping
AM08	Amhara - North Wollo - Bugna	10 - 2010	1	480	mild	mild	14.6	82.2	crisis	cropping
AM09	Amhara - South Wollo - Dessie Zuria	04 - 2010	2	465	normal	normal	11.3	83.5	crisis	cropping
AM10	Amhara - South Wollo - Dessie Zuria	07 - 2010	0	257	normal	normal	11.9	87.1	stressed	cropping
AM11	Amhara - North Gondar - East Belesa	04 - 2010	3	672	normal	mild	18.8	52.1	crisis	cropping

AM12	Amhara - North Wollo - Lasta	04 - 2010	5	641	normal	moderate	13	85.9	stressed	cropping
AM13	Amhara - South Wollo - Mekdela	06 - 2010	2	561	mild	moderate	15.7	77.2	stressed	cropping
AM14	Amhara - North Gondar - Wegera	11 - 2010	0	521	mild	mild	13.4	76.4	crisis	cropping
AM15	Amhara - North Wollo - Delenta	08 - 2010	2	366	normal	normal	11.1	89.3	stressed	cropping
AM16	Amhara - South Wollo – Tenta	04 - 2010	1	859	normal	normal	7.4	88.7	crisis	cropping
BG01	Benishangul-Gumuz - Asosa - Bambasi	11 - 2010	4	590	severe	severe	12.5	78.5	minimal	cropping
BG02	Benishangul-Gumuz - Asosa - Kurmuk	12 - 2010	3	520	severe	severe	17.1	83.8	stressed	cropping
DD01	Dire Dawa - Dire Dawa - Dire zuriya	08 - 2010	1	510	normal	mild	13.1	65.0	NA	agropastoral
OR08	Oromia - West Harerghe - Darolebu	10 - 2010	2	553	normal	normal	5.5	67.1	crisis	agropastoral
OR09	Oromia - Borena - Gelana	12 - 2010	0	577	mild	mild	5.7	69.2	crisis	agropastoral
OR10	Oromia - East Harerghe - Kersa	03 - 2010	1	709	normal	normal	7.6	50.2	stressed	cropping
OR11	Oromia - East Harerghe - Melkabello	11 - 2010	1	610	normal	normal	9.5	66.9	crisis	cropping
OR12	Oromia - Bale - Mena Angetu	02 - 2010	6	731	normal	moderate	17.7	44.3	stressed	cropping
OR13	Oromia - Bale - Mena Angetu	07 - 2010	5	794	normal	normal	5.5	54.6	stressed	cropping
OR14	Oromia - East Harerghe - Meta	02 - 2010	4	721	mild	severe	10.7	77.3	stressed	cropping
OR15	Oromia - East Harerghe - Meta	10 - 2010	1	679	normal	mild	9.7	71.7	crisis	cropping
OR16	Oromia - Borena – Abaya	02 - 2010	2	737	normal	moderate	10.1	35.8	stressed	agropastoral
OR17	Oromia - West Harerghe - Boke	01 - 2010	2	674	mild	moderate	15.4	44.0	stressed	agropastoral
OR18	Oromia - West Harerghe - Habroo	02 - 2010	0	644	normal	mild	10.4	44.4	stressed	cropping
SN10	SNNP - Sidama - Boricha	06 - 2010	1	508	severe	severe	5.5	79.1	crisis	cropping
SN11	SNNP - Wolayita - Damot Gale	03 - 2010	0	702	normal	mild	4.9	71.4	stressed	cropping
SN12	SNNP - Wolayita - Damot Pulassa	06 - 2010	2	545	mild	mild	5.1	82.2	stressed	cropping
SN13	SNNP - Wolayita - Offa	02 - 2010	4	622	normal	mild	8.2	47.0	stressed	cropping
SN14	SNNP - Sidama - Shebdino	03 - 2010	2	699	normal	mild	9.9	77.5	stressed	cropping
SN15	SNNP - Wolayita – Humbo	04 - 2010	3	933	mild	mild	4.0	91.0	crisis	cropping
AM17	Amhara - North Gondar - East Belesa	04 - 2011	1	697	normal	mild	13.8	72.1	crisis	cropping
AM18	Amhara - North Wollo - Habru	01 - 2011	2	716	severe	severe	4.9	73.6	stressed	cropping
AM19	Amhara - Wag Hamra - Sekora	03 - 2011	0	609	normal	mild	14.5	80.7	stressed	cropping
AM20	Amhara - North Gondar - Wegera	12 - 2011	1	690	mild	mild	10.6	11.9	stressed	cropping
DD02	Dire Dawa - Dire Dawa - Dire zuriya	05 - 2011	4	583	severe	severe	12.0	57.4	stressed	agropastoral
OR19	Oromia - West Harerghe - Boke	01 - 2011	2	650	moderate	normal	6.7	82.8	crisis	agropastoral
OR20	Oromia - Borena - Bule Hora	02 - 2011	1	765	moderate	severe	12.3	36.7	stressed	agropastoral

OR21	Oromia - West Hareghe - Habroo	02 - 2011	0	460	mild	normal	10.2	66.4	stressed	cropping
OR22	Oromia - East Harerghe - Kurkfa Chelle	01 - 2011	2	506	normal	normal	8.8	79.6	stressed	cropping
OR23	Oromia - Guji - Liben	04 - 2011	2	1007	severe	severe	14.4	78.3	crisis	agropastoral
OR24	Oromia - East Harerghe - Melkabello	12 - 2011	1	629	normal	normal	9.3	86.6	stressed	cropping
OR25	Oromia - East Harerghe - Meta	10 - 2011	0	754	mild	severe	10.5	8.2	stressed	cropping
OR26	Oromia - East Harerghe - Midega Tola	01 - 2011	2	536	normal	normal	11.9	56.7	crisis	cropping
OR27	Oromia - West Harerghe - Oda Bultum	03 - 2011	1	558	mild	mild	12.6	61.7	stressed	cropping
AM21	Amhara - South Wollo - Dessie Zuria	03 - 2012	1	490	normal	normal	13.6	81.5	stressed	cropping
AM22	Amhara - Wag Hamra - Ziquala	09 - 2012	6	709	normal	normal	13.7	67.7	crisis	cropping
OR28	Oromia - Borena - Abaya	02 - 2012	0	622	mild	moderate	5.6	6.1	minimal	agropastoral
OR29	Oromia - Borena - Bule Hora	02 - 2012	0	710	moderate	severe	5.0	2.1	crisis	agropastoral
OR30	Oromia - West Harerghe - Oda Bultum	03 - 2012	0	567	moderate	mild	4.8	11.6	stressed	cropping
SN16	SNNP - Sidama - Boricha	05 - 2012	2	748	moderate	moderate	8.2	5.8	stressed	cropping
SN17	SNNP - Wolayita - Damot Pullasa	09 - 2012	3	538	normal	mild	4.1	93.3	crisis	cropping
AF01	Afar - Afar Zone 4 - Gulina	05 - 2013	4	589	severe	severe	11.3	8.1	stressed	pastoral
AF02	Afar - Afar Zone 2 - Koneba	04 - 2013	4	625	normal	normal	11.7	28.5	crisis	agropastoral
AF03	Afar - Afar Zone 2 - Megale	05 - 2013	3	664	mild	mild	11.4	31.9	crisis	pastoral
OR31	Oromia - West Harerghe - Anchar	09 - 2013	1	769	normal	normal	10.4	63.0	crisis	agropastoral
OR32	Oromia - Borena - Bulehora	03 - 2013	2	661	moderate	severe	3.3	54.0	stressed	agropastoral
OR33	Oromia - West Harerghe - Darolebu	04 - 2013	0	698	mild	mild	6.4	60.1	crisis	agropastoral
OR34	Oromia - East Harerghe - Deder	09 - 2013	1	802	normal	normal	8.0	67.5	stressed	cropping
OR35	Oromia - East Harerghe - Fedis	10 - 2013	1	794	normal	normal	8.0	91.4	stressed	cropping
OR36	Oromia - East Harerghe - Meta	11 - 2013	1	687	mild	severe	5.8	81.9	stressed	cropping
OR37	Oromia - West Harerghe - Oda Bultum	03 - 2013	0	606	mild	mild	4.9	60.2	crisis	cropping
SN18	SNNP - Wolayita - Damot Pulassa	02 - 2013	1	476	mild	mild	4.1	79.3	stressed	cropping
AF04	Afar - Afar Zone 2 - Afdera	04 - 2014	4	575	normal	normal	27.0	4.6	crisis	pastoral
AF05	Afar - Afar Zone 4 - Gulina	07 - 2014	1	522	normal	normal	11.6	12.2	stressed	pastoral
GA01	Gambella - Nuer - Jikawo	08 - 2014	14	588	mild	severe	18.4	56.0	minimal	cropping
OR38	Oromia - Borena - Bule Hora	03 - 2014	2	606	normal	normal	5.3	49.0	minimal	agropastoral

The mortality, malnutrition, and vaccination coverage data were obtained from the small-scale mortality and nutrition surveys from the Complex Emergency Database (CE-DAT). The short- and long-term droughts in a given location were based on the 3- and 12-month standardized precipitation evapotranspiration index (SPEI), respectively, and obtained from the Global Drought Monitor database, and then categorized as no drought (SPEI > 0), mild drought (–1 < SPEI ≤ 0), moderate drought (–1.5 < SPEI ≤ –1) and severe drought (SPEI ≤ –1.5). The food insecurity and livelihood zones data were obtained from the Famine Early Warning Systems Network (FEWS NET). NA: data not available.
